# Can too much exercise kill you? A systematic review of the risk of a cardiovascular event or death from long term strenuous exercise

**DOI:** 10.1007/s10654-025-01353-3

**Published:** 2026-01-24

**Authors:** Aleksandra Saulicz, David Abernethy, Darren Wraith

**Affiliations:** 1 School of Public Health & Social Work, Queensland University of Technology (QUT), 149 Victoria Park Road, Kelvin Grove, QLD 4059 Australia; 2 School of Exercise and Nutrition Sciences, Queensland University of Technology (QUT), Kelvin Grove, QLD Australia; 3 Viertel Research Centre, Cancer Council Queensland, Brisbane, QLD Australia

**Keywords:** Vigorous exercise, Physical activity, Acute cardiovascular events, Exposure-response relationship, Public health, Epidemiology

## Abstract

A considerable number of studies have highlighted that engaging in physical activity (PA) improves health and reduces risk of mortality. But is there a limit? Some evidence suggests a U-shaped exposure-response in which the protective benefits of strenuous exercise eventually diminish (at some point) and the risk of a cardiovascular event or death increases, thereby questioning a linear association between exercise volume, intensity, and cardiovascular events/mortality. This review evaluates the evidence and critically appraises studies assessing whether strenuous exercise is associated with increased cardiovascular-related events from an epidemiological perspective. After a comprehensive literature search, 1,908 records were identified; 1,068 remained after deduplication. Title/abstract screening excluded 950 records; 66 full texts were assessed; 14 studies were included for critical review. Two independent assessors appraised study quality and risk of bias, focusing on exposure and outcome measurement, confounder control, and whether timeframes and follow-up were adequate to observe associations. Evidence supporting a U-shaped exposure-response was limited. No study achieved the highest quality rating. Common limitations included inconsistent intensity classification, absence of repeated exposure measurement, inconsistencies with outcome measures, insufficient follow-up to capture long-term effects, and incomplete adjustment for key confounders. The current evidence supports the benefits of regular PA, including strenuous activity, with benefits appearing to plateau at very high doses. Further rigorous epidemiological research with repeated exposure measures, standardised outcomes, adequate follow-up, and more robust control for confounding is needed.

**Supplementary Information:**

The online version contains supplementary material available at 10.1007/s10654-025-01353-3.

## Introduction

 Physical activity (PA) has been recognised as a significant indicator of an individual’s overall health status, regardless of age, as well as a major modifiable risk factor for non-communicable disease and mortality [1–4]. A myriad of literature has analysed the relationship between PA and cardiovascular health specifically, indicating that regular engagement in moderate-to-vigorous physical activity (MVPA) can help lower the risk of cardiovascular disease (CVD), coronary heart disease events and mortality [3, 5–7]. Some evidence from a recent longitudinal cohort study suggests that the lowest risks of cardiovascular events are present among individuals that exercise most [8]. Furthermore, multiple studies have proposed that increased PA levels, regardless of intensity or metabolic equivalent (MET), are beneficial to health and longevity [3, 5, 9–13]. This wealth of epidemiological evidence has helped to inform current adult PA World Health Organisation guidelines [14] that recommend engaging in at least 150–300 min of moderate-intensity aerobic activity (3 to 5.9 Metabolic Equivalent Tasks (METs)) or at least 75–150 min of vigorous-intensity activity (> 6 METs) or a combination of the two per week. Further uptake of PA is recommended to yield additional benefits as well as to engage in muscle-strengthening activities [14]. Despite the extensive literature on the health benefits of moderate and vigorous PA, far less is known about the cardiovascular implications of sustained high-intensity and high-volume exercise. Additionally, contradicting evidence exists pertaining to the ideal volume and intensity of PA needed to yield the greatest benefits for cardiovascular health and mortality.

Limited evidence has suggested that the proposed health benefits do not apply to individuals that perform high-intensity and high-volume exercise (high PA levels), particularly in acute timeframes [15, 16]. However, further studies that have identified this association were only able to do so in individuals with underlying conditions or cardiovascular risk factors [17–19]. For this review, strenuous PA refers to exercise performed at the upper-end of both intensity and overall volume, reflecting a combination of vigorous-to-very-high effort and substantial accumulated duration or workload. While recent recommendations by Bishop et al. (2025) provide clearer guidance on defining higher-intensity exercise domains [20], their classifications alone do not capture the large weekly doses of PA typically undertaken by highly active individuals. Accordingly, strenuous PA in this review includes activities that are not only physiologically demanding but also performed in considerable quantities over time, such as regular long-distance running, cycling, rowing, cross-country skiing, and swimming. These patterns represent the extreme upper limit of both PA intensity and volume in the general population. Framing strenuous PA in this way allows a more accurate assessment of whether very high doses of PA continue to yield cardiovascular benefits or whether they may approach a point of diminishing returns.

**Fig. 1:**
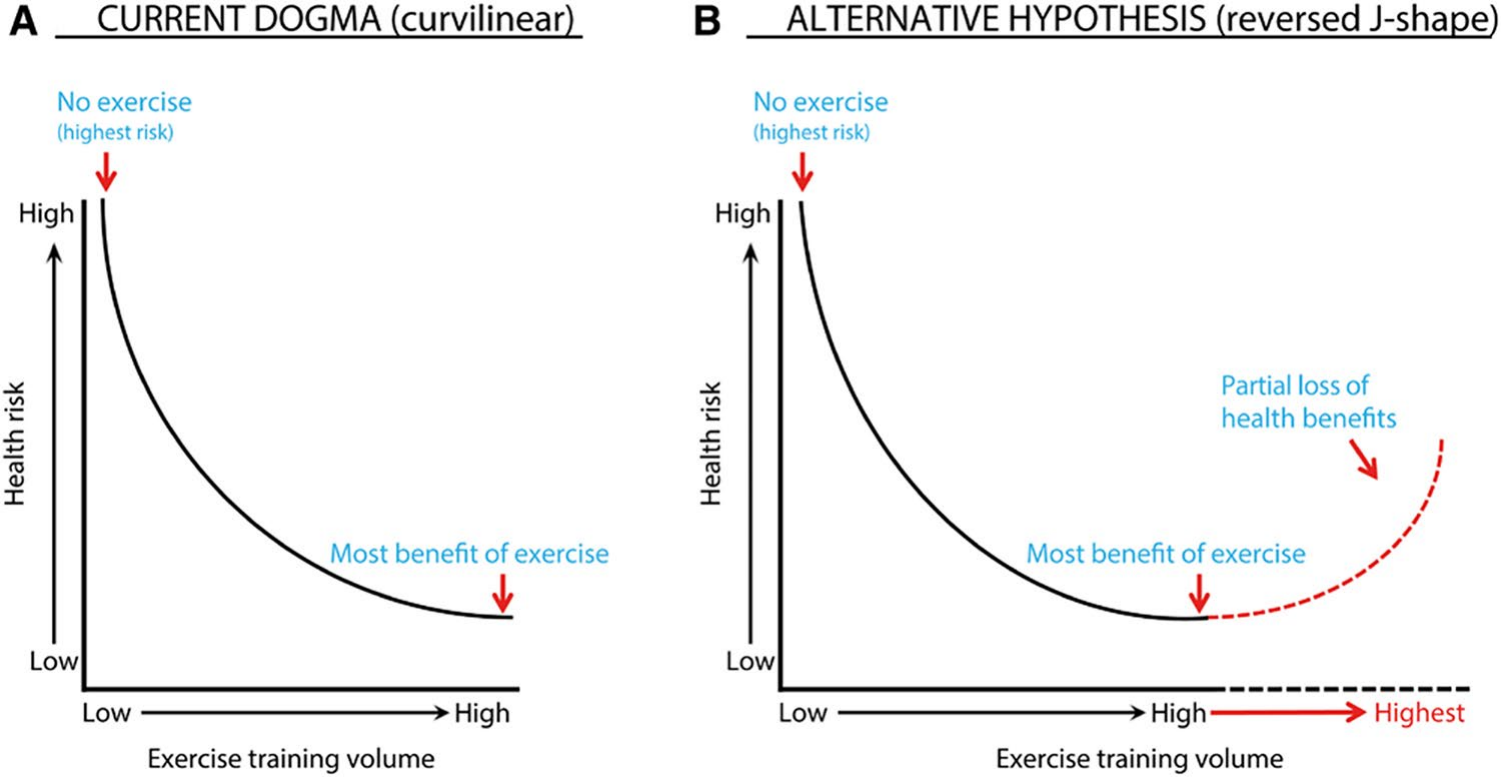
Conceptual overview of dose-response association between physical activity volume and cardiovascular health outcomes [8, 21]. Conceptual overview of dose-response association between physical activity volume and cardiovascular health outcomes in line with **A** the current dogma and ***B*** an alternative hypothesis. Source: AHA Statement and adapted from Eijsvogels et al. [21] Copyright © 2018, The Authors. This article is distributed under the terms of the Creative Commons Attribution 4.0 International License (http://creativecommons.org/licenses/by/4.0/), which permits unrestricted use, distribution, and reproduction in any medium, provided you give appropriate credit to the original author(s) and the source, provide a link to the Creative Commons license, and indicate if changes were made

The uncertainties in the current evidence base have led several researchers to hypothesise potential mechanisms that might explain increased cardiovascular risk at very high doses. A J or U-shaped exposure-response has been proposed where the protective benefits of higher levels of PA (at some point) start to reduce and the risk of a cardiovascular event increases (or risk of death in some instances) [21–23] (see Fig. 1). O’Keefe et al. [22] suggest that long-term excessive endurance exercise may induce pathologic structural remodelling of the heart and large arteries, highlighting that while moderate exercise is beneficial for cardiovascular health, excessive endurance exercise may lead to adverse cardiovascular effects, including increased risks of atrial fibrillation and coronary artery calcification. Some reports on elite athletes point out the increased risk of having heart issues, such as a study by Harmon et al. [24] which demonstrates that the incidence of sudden cardiac death (SCD) in National Collegiate Athletic Association (NCAA) athletes is significantly higher than previously estimated. Regular high-volume endurance training imposes increased hemodynamic demands that alter the loading conditions of the heart, particularly among athletes participating in sports requiring sustained elevations in cardiac work (e.g.long-distance running, rowing, swimming, cycling) [25]. Some of the remodelling that occurs in endurance athletes may not be entirely benign, as in elite athletes, cardiac dimensions do not completely regress to normal levels even several years after the athlete has retired from competition [22].

Eijsvogels et al. [21] found limited circumstantial evidence supporting the reverse J or U-shaped exposure-response relationship to exist particularly at high PA levels but concluded that further studies are needed to establish the association. Mittleman et al. [26] summarised the uncertainty of the benefits of high PA levels by concluding that it can both trigger and prevent myocardial infarction, acting as a double-edged sword [26].

Given these mixed physiological and epidemiological findings, it is important to evaluate whether current evidence is sufficient to clarify the cardiovascular effects of strenuous PA performed over long periods of time. Previously, only two meta-analyses and systematic reviews [9, 27] have investigated the association of strenuous PA and mortality risk in general populations; incorporating mortality from all causes, CVDs, cancer, and coronary heart disease. Both studies focused predominantly on the statistical analysis of the combined results included in their review, as opposed to a critical or quality assessment of those studies. The study by Rey Lopez et al. [27] also only considered studies which examined vigorous versus moderate intensity which led to a relatively small number of studies being examined (*n* = 5). As there can be a long time between the exposure (high PA) and the outcome (e.g. cardiovascular event) many of the studies rely on observational cohort data which can be problematic in terms of issues relating to missing data, accuracy of measurements, various types of bias, and the influence of confounding factors to name only some. Consequently, the purpose of our systematic review is to critically assess the quality of the current literature (accounting for key confounding variables, accurate and repeated measurements of exposure/outcomes, etc.) for the general physically active population regularly engaging in strenuous PA and present the overall findings to provide recommendations for future research. These findings are critically important to provide some reliable evidence to a large section of the community who engage in this type of activity and to provide clearer public health or health promotion messages about the health benefits of physical exercise.

## Methods

This systematic review was designed according to the Preferred Reporting Items for Systematic Review (PRISMA) guideline [28]. The review followed the recommendations on data searching and data processing as described in the Cochrane Handbook for Systematic Reviews, Chap. 7 [29]. The population, exposure, reference group, and outcome were defined as follows:

- Population: General population.
  This review analysed the studies conducted on the general population, with most of the studies located in Australia, US, UK, Scandinavia, and other European countries. However, geographical location was not a result of inclusion criteria but rather these regions provided a useful source of data due to popularity of cardiovascular-related activities (e.g.: jogging).
- Exposure: Strenuous PA* performed regularly over a number of years (> 5 years).
  Studies were included only if they examined participants engaging in PA at the high-to-very-high end of both intensity and volume. While definitions varied across studies, strenuous PA in this review was classified as PA of vigorous intensity and was performed at this level over a number of years. Studies that assessed general PA without focusing on this high intensity/high-volume threshold were excluded.
- Reference group: Low and moderate levels of physical activity or non-exercisers.
- Outcome: Cardiovascular event or related death.

A meta-analysis was not conducted, as the included studies were highly heterogeneous in their exposure definitions, outcome measures, follow-up durations, and confounder adjustments. Meta-analysis is appropriate only when studies are sufficiently exchangeable, that is, when they involve comparable populations, exposures, outcomes, and methods [30] and this was not the case for the present evidence base. These methodological differences made statistical pooling of results inappropriate and misleading; therefore, a narrative synthesis was undertaken to summarise and critically appraise the evidence.

### Search strategy, study selection and data extraction

Both, medical subject heading (MeSH) terms and natural language expressions were combined for the search in electronic databases. Medline EBSCOHost, PubMed, Embase and Scopus were independently searched for relevant literature. Keywords included in the searches include physical activity*, exercise*, strenuous activity*, vigorous activity*, mortality*, dose response*, cardiovascular*, heart disease*. Citation chaining was used to help supplement search results.

Inclusion criteria used for potential studies included those with an observational design, general populations as their sample, PA as the main exposure variable and mortality, heart arrhythmias or other physical exercise related heart conditions, acute cardiovascular events as the outcome variable. Exclusion criteria included studies with irrelevant PA data, an irrelevant outcome (non-cardiovascular/mortality), drug trials, reviews, commentary pieces, studies published in languages other than English, studies without an abstract or duplicates. For further details see Table 1 below.

**Table 1:** Selection criteria

Item	Inclusion Criteria	Exclusion criteria
Design	Observational studies	N/A
Population	General adult population	More clinically focused studies targeting only professional athletes
Condition	Sufficient follow up period, reports PA dimensions (MET or dose of exercise performed in a week)	No reports on PA categories, too short timeframe, not focused on strenuous intensity
Outcome	Mortality, all-cause mortality, heart arrhythmias or other physical exercise related heart conditions, acute cardiovascular events, CVD/CVRFs	All-cause mortality not an outcome; conditions not associated with CVD

N/A indicates not applicable

CVD = Cardiovascular Disease, CVRFs = Cardiovascular Risk Factors, MET = Metabolic Equivalent, PA = Physical Activity

### Search results - PRISMA flowchart

See Fig. 2.

**Fig. 2:**
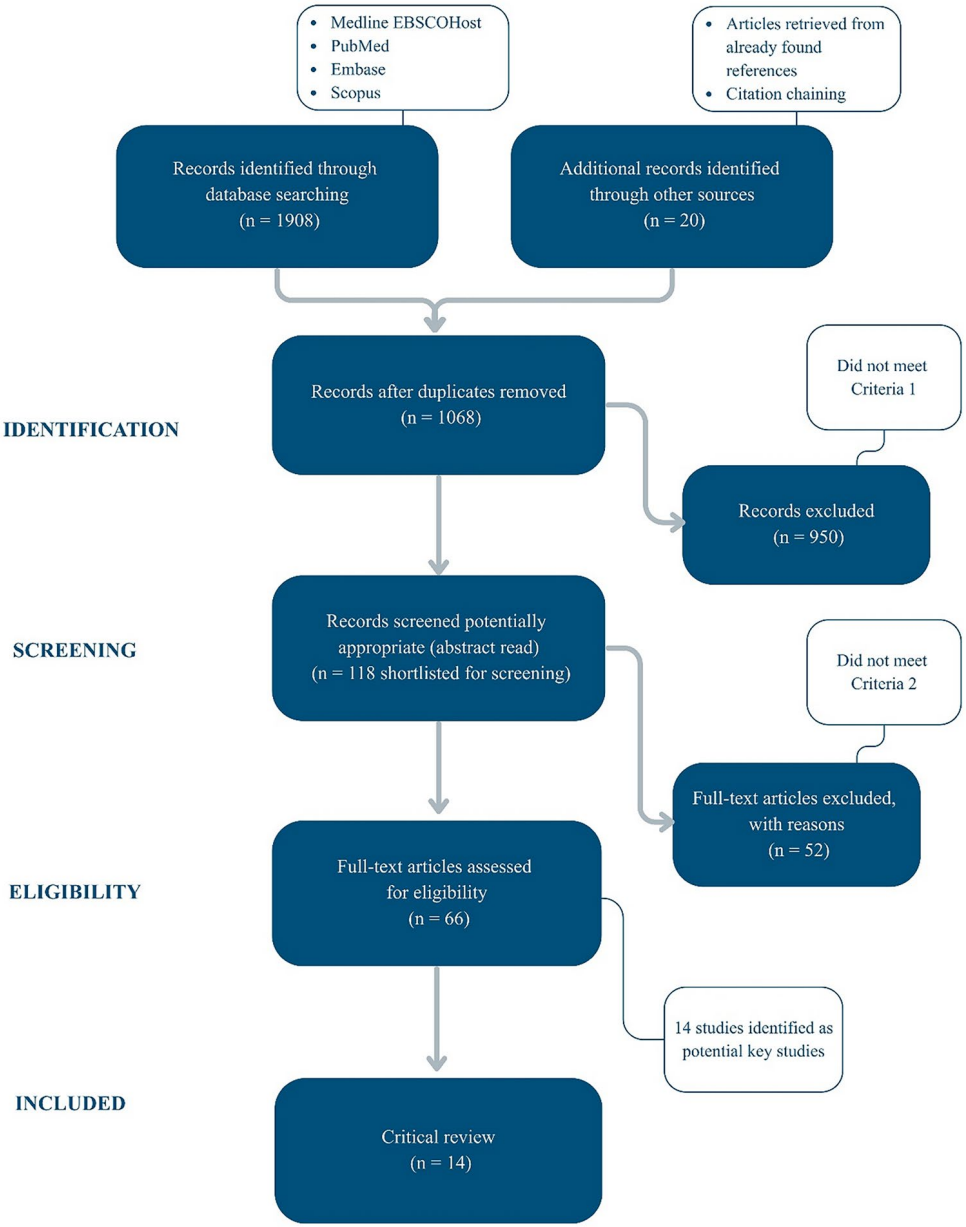
Search results - PRISMA flowchart

### Quality assessment

This review utilised the National Institute of Health (NIH) [31] quality assessment tool for observational cohort and cross-sectional studies. This tool was selected due to its extensive list of questions, designed to assist with reviews and concepts related to internal validity. The NIH tool can be found in the Supplementary Materials.

The overall quality of the fourteen studies in this review varied, out of a total of fourteen points, scores ranged from 6 [32], 7 [33, 34], 8–8.5 [35–40] and 9–10 [23, 41–44]. The critical appraisal criteria and scoring system with final scoring and quality can be found in the Supplementary Materials. Many studies bordered between the poor, fair and good overall cut-offs, highlighting their limitations. The potential for the results from these studies being misinterpreted is somewhat large. A critical appraisal of the likelihood of bias for the fourteen included studies was undertaken, specifically to examine the likelihood of bias due to issues with exposure, outcome, timeframe/follow-up, and confounding variables.

### Risk of bias assessment

Comprehensive risk of bias for all reviewed studies was appraised by two independent assessors (AS and DA), as recommended by the Cochrane Handbook [29], to ensure the validity and reliability of the findings. Any discrepancies in the scoring were resolved through discussion or consultation between the two assessors (AS and DA) and, in case of further disagreement, a third reviewer (DW) was included in a final judgement. The results of the risk of bias assessment were categorized as low risk (green), probably low risk (lighter yellowish green), moderate risk (yellow), probably high risk (orange) or high risk (red) for each domain. These assessments are presented in Table 3 in the results section, providing a detailed account of the potential biases and their implications for the study results. This rigorous assessment helps to highlight the strengths and limitations of the included studies, ensuring a transparent and robust evaluation of the evidence.

The domain of ‘exposure assessment methods’ was characterized by the least risk of bias across all evaluated studies (see Risk of Bias Table 3). Only three studies were evaluated as high risk of bias amongst fourteen appraised studies within these domains. Over half of the studies (nine) had a moderate to high risk of bias in terms of adequate timeframe to observe the association between the exposure and outcome. In the domain related to addressing confounders, the studies were assessed as moderate risk (six studies) and probably high risk (eight studies). For the assessment of overall bias of each study, four studies stand out as overall being of lower risk [38, 39, 41, 42], and this is somewhat consistent with the results from the quality assessment, as these studies scored between 8.5–9.5 (borderline fair-good/good).

Additionally, the certainty of evidence for the studies included in this review was evaluated using the Grading of Recommendations Assessment, Development and Evaluation (GRADE) approach. GRADE is a structured framework employed in systematic reviews and meta-analyses to assess the certainty of evidence across included studies, taking into account domains such as risk of bias, inconsistency, indirectness, imprecision, and publication bias, providing an overall rating of high, moderate, low or very low confidence in the effect estimates [45].

In accordance with the GRADE framework, the certainty of evidence for all studies was low as all included studies were observational in design. Although notable concerns were identified during our risk of bias assessment, the evidence base did not exhibit a level of bias to warrant further downgrading to very low, and the remaining GRADE domains (inconsistency, indirectness, imprecision and publication bias) could not be reliably evaluated in the absence of a meta-analysis.

## Results

There were 1908 records initially identified, with 1068 remaining after the removal of duplicates. Screening of titles and abstracts excluded 950 articles, with 66 shortlisted for full-text screening. A total of 14 articles were included for critical evaluation. The literature search and subsequent screening procedure is illustrated in Fig. 2, visualizing the study selection process according to the PRISMA guidelines.

Twelve (85.7%) were cohort studies, as for the remaining two (14.3%), one was a repeated survey cross-sectional [37] and the other a single timepoint cross-sectional study [35]. All studies in this review assessed PA using self-reported measures, and the outcomes of interest encompassed CVD, all-cause mortality, and cardiovascular-related mortality. Further descriptions of each study included in this review are exhibited in Table 2.

**Table 2:** Main characteristics of prospective studies (*n* = 14) identified in the systematic review

Study	Quality Assessment (NIH score)	Study design	Population size	Region	Duration	Methodology	Measures and PA classification	Key Findings
Aadahl et al. [35]	Fair (8.5)	Cross-sectional	1,693	Danish population, both men and women, aged 33–64.	3 years follow up	Self-reported questionnaire to measure association between activity level and biological variables.	PA measured in METs score (≤ 45 METs and > METs per 24 h) and 4-way activity classification; mortality was not an outcome of measure; accounted for all relevant confounding variables.	Findings suggest possible threshold of benefits in the association between PA and vital cardiovascular biomarkers (linear association between overall PA and BMI, waist circumference, waist hip ratio, HDL, and triglycerides with PA level up to 45 METs, no longer linear above that level).
Arem et al. [41]	Fair (9)	Cohort	661,137 (6 cohorts)	US and Swedish population aged 21–98.	14.2 years follow up	Self-reported physical activity; BMI and mortality analysis.	Measured in MET (≥ 6.0 METs); accounted for some of the relevant confounding variables.	There is no indication of increased mortality risks associated with leisure time physical activity levels as high as 10 times or more of the recommended minimum. The greatest mortality benefits can be found at the moderate-intensity activity levels.
Brown et al. [33]	Fair (7)	Cohort	18,748	Australian population, both men and women, aged 65–83 and 70–75 respectively.	13 years follow up	Self-reported, behavioural, socio-demographic, and health characteristics.	Measured in MET (high 1050-<1500; very high ≥ 1500). Accounted for most of the relevant confounding variables.	There is a dose-response relationship between all-cause mortality and levels of physical activity in elderly of either gender and there are benefits found of low level of exercise in both genders (risk reduction greater in woman for any given activity level).
Bucksch [42]	Fair/Good (9.5)	Cohort	7,187	German population aged 30–69.	16 years follow up	Self-administered leisure time physical activity questionnaire.	Measured as ≥ 6 METs (vigorous); linear relation between PA overall volume and all-cause mortality risk reduction; accounted for most of relevant confounding variables.	The risk of all-cause mortality could be seen to be reduced solely in female group and in a moderate intensity of activity. For men, only vigorous activity found a clear risk reduction. Conflicting findings may suggest imprecise measurements and/or different characteristics of participants.
Gebel et al. [34]	Fair (7)	Cohort	204,542	Australian population, aged 45–75.	Mean 6.52 years follow up	Self-reported questionnaire.	Calculated vigorous activity MVPA by vigorous activity x2/weighted MVPA x 100%. Measured 3 categories of MVPA for vigorous activity; did not account for most of relevant confounding variables.	Inverse dose-response relationship with all-cause mortality in vigorous group. Higher-intensity activities provide more cardio-protection.
Janssen & Jolliffe [36]	Fair (8.5)	Cohort	1,045	US population with coronary artery disease aged ≥ 65years.	9 years follow-up	Interview and clinical examination.	High end group measured > = 4 mph; did not account for most of relevant confounding variables.	Curvilinear relationship between physical activity level and all-cause mortality risk in dose-response manner (for population with CAD). Vigorous activity did not lead to additional benefits beyond that of low or moderate intensity (and mortality was not as low as in the remaining groups).
Joseph et al. [43]	Good (10)	Cohort	19,329	Danish population (men and women) aged 20–98.	From date of first examination (’76-’78) till 2018 or death. Mean follow up time of 23.4 ± 11.7 years.	Four examinations with new random sample were included each time (from original sample). Self-administered questionnaire and clinical examination.	PA measured by > 4 h/wk. or regular heavy exercise or competitive sports several times a week; high intensity expressed in > 6 METs; accounted for only some of relevant confounding variables.	Inverse dose-response association confirmed between physical activity and all-cause mortality (at all levels of blood pressure), PA associated with reduction in cardiovascular-related events irrespectively of PA level. However, due to very low number in the highest-intensity spectrum, for the analysis the variables were categorised into combined moderate-high level activity.
Loprinzi [37]	Fair (8)	Prospective study with cross-sectional survey	16,049	US population from NHANES, aged 18–85.	8–9 years follow up	Assessed 48 different individual PAs, open-ended questionnaire, and cardiovascular biomarkers.	Measured min MVPA (min 300 min/wk.) MET-min-month, calculated by multiplying number of days by the mean duration by the respective MET level (MET-min-month = days × duration × MET level); did not demonstrate great survival effects; did not account for most of relevant confounding variables.	No evidence of harmful effect of very high activity, however protective effect on mortality was reduced when compared with lower levels.
Maessen et al. [38]	Fair (8.5)	Cohort	21,266	Dutch population, aged ≥ 35 years.	Measured over a median of 32 years	Online questionnaire, analysis of lifelong exercise patterns, history of CVD, lifestyle factors.	Measured as approx. 350-minute run at approx. 8 km/h on a weekly basis (w8.5 MET - vigorous); did not account for most of relevant confounding variables.	Demonstrated curvilinear association between cardiovascular morbidity and lifelong exercise patterns. Higher doses of exercise do not yield additional health benefits; best results found at the low exercise doses.
Oja et al. [39]	Fair (8.5)	Cohort	80,306	British population, both men and women, aged 30–98.	9.2 ± 4.5 years follow up	Self-measured; study questionnaire and measurements of height and weight.	Non-occupational PA measured by frequency, duration of participation and intensity (MET-hours/week calculated using PA Compendium); accounted for most of relevant confounding variables.	Some indication of U-shaped dose–response relationships between some activities and mortality (cycling, swimming, racquet sports).
Schnohr et al. [23]	Fair (9)	Cohort	5,048	Danish population (Copenhagen) both men and women, aged 20–93.	12 years follow up	Original random sample follow ups and new random sample, CV epidemiological surveys (excluded those with history of CHD, stroke, or cancer).	PA measured by > 4 h/wk. or regular heavy exercise or competitive sports several times a week; notes that even slow jogging (6METs) corresponds to vigorous exercise, and strenuous jogging corresponds to very vigorous exercise (≥ 12 METs); accounted for most of relevant confounding variables.	Suggests U-shape association between all-cause mortality and dose of jogging. Light and moderate group appears to benefit more than strenuous group.
Kikuchi et al. [44]	Fair/good (9.5)	cohort	83,454	Japanese population, 40–69 years of age	12 years follow up	Self-reported questionnaire at baseline and 2 follow ups (after 5 & 10 years), high follow up rate (76%)	PA measured by frequency and duration, assigned METs of 3.0 and 6.0 to MPA and VPA respectively; all-cause mortality considered; not accounted for some confounding variables.	Suggests comparable health benefits at both moderate PA and vigorous PA. Self-reported measure may cause misclassification between MPA and VPA.
Lahti et al. [32]	Poor (6)	Cohort	13,346 (7,148 women and 1,799 men)	Finnish population from Helsinki Health Study 40–60 years old	A mean of 12 years	Self-reported questionnaire, 67% response rate at baseline, measured once at baseline	PA measured in METs score. Significant difference between men and women sample size population - younger people, men and manual workers underrepresented. Study cohort is relatively active physically compared with European adults in general. Not accounted for diet in confounding variables.	Vigorous PA associated with reduced mortality risk independently of the volume of leisure PA and other covariates.
Shiroma et al. [40]	Fair (8.5)	Cohort	46,650 (38,671 women and 7,979 men)	7979 men from Harvard Alumni Health Study (HAHS) and 38,671 women from Women’ Health Study (WHS)	A mean follow-up of 17.3 years in men and 16.4 years in women	Self-reported questionnaire	PA measured in METs score; Significant difference between men and women sample size population	For men: vigorous PA further reduces all-cause mortality rate but not the CVD rate compared to moderate PA. For women: moderate PA to be at least as beneficial as vigorous PA when MVPA volume is same (potential for residual confounding by diet and other healthy habits in that group).

BMI = Body Mass Index, CAD = Coronary Artery Disease, CV = Cardiovascular, CVD = Cardiovascular Disease, HDL = High-Density Lipoprotein, METs = Metabolic Equivalents, MVPA = Moderate-to-Vigorous Physical Activity, NHANES = National Health and Nutrition Examination Survey, PA = Physical Activity, US = United States

For the studies utilising cross-sectional methods, Aadahl et al. [35] investigated the exposure-relationship between PA and cardiovascular related risk factors, with a range of biological factors and anthropometric measures as outcome variables. The results highlighted a linear association between overall PA level and CVD risk factors. However, there was no linear association with an activity level above 45 METs. After a three-year follow-up incorporating self-reported 24-hour MET scores, a linear association was confirmed between overall PA and BMI, waist circumference, waist hip ratio, HDL and triglycerides. Loprinzi [37] examined the association between self-reported MVPA and both cardiovascular biomarkers and mortality. After completing Cox proportional hazards regression, no evidence was present to suggest that very high MVPA levels were harmful to health. The study found that even engaging in MVPA below the minimum recommendation yielded survival benefits, with the greatest benefits observed at five times the minimum recommendation. Although very high MVPA levels (ten times the minimum recommendation) in this study did not demonstrate any harmful effects, they were not associated with the greatest benefits.

The remaining studies in this review were all cohort studies, conducted across different geographical contexts. Two studies in this review incorporated Australian data to examine the relationship between PA and all-cause mortality. Gebel et al. [34] examined this relationship using 45 and Up data. The authors noticed an inverse dose-response relationship between total vigorous PA minutes (independent of total MVPA) and mortality, highlighting a protective benefit of vigorous PA. Brown et al. [33] analysed a similar relationship in older adults. It was noted that risk reductions were between 30% and 50% higher in women than men in each of the individual PA categories used in the study, further highlighting the exposure-response relationship between all-cause mortality and varying levels of PA. Janssen & Jolliffe [36] incorporated a sample of U.S. adults with coronary artery disease to examine the same relationship as the two previous studies. Their findings further corroborated the concept of a curvilinear relationship between baseline leisure PA and risk of all-cause mortality, where a plateau in mortality risk occurred at 4000 kcal per week. Arem et al. [41] conducted a study to determine the upper limits of mortality benefits from high intensity PA, analysing leisure activities with corresponding MET values. From their combined U.S. and Swedish sample of adults (21–98 years), physically active individuals had a lower risk of adverse cardiac events and those engaging in activities for more than 75 MET hours/week had a further reduction in mortality risk. Another study on a U.S. population by Shiroma et al. [40], concluded that for men, vigorous PA further reduces all-cause mortality rate but not the CVD rate when compared to moderate PA. On the other hand, for women, moderate PA turned out to be at least as beneficial as vigorous PA when MVPA volume is the same (noting the potential for residual confounding by diet and other healthy habits in that group). Kikuchi et al. [44] in their prospective cohort study of a Japanese population, suggested comparable health benefits for both moderate PA and vigorous PA as long as individuals meet guidelines, however the number of subjects categorised in engaging in VPA was low.

The final six studies included in this review were conducted in varying European samples. Bucksch [42] also examined the relationship between PA and all-cause mortality, using a German cohort. A linear relation between overall volume of exercise and all-cause mortality risk reduction was present, with both moderate and vigorous intensity exercise categories (determined by MET scores) incorporated in the analysis. The results of this study indicated that there was a clear inverse association between total PA volume and all-cause mortality (particularly in women). Engaging in at least 2.5 h of moderate-intensity activity was associated with a reduced risk of overall mortality. Benefits appeared to plateau for men, as the influence of lifestyle activities were less pronounced compared to women. Maessen et al. [38] further explored the conceptual curvilinear relationship between PA and cardiovascular morbidity and risk factors, displaying similar results in a sample of Dutch adults (≥ 35 years). The lifelong approach concluded that the prevalence of CVD and relevant risk factors was lower for each exercise quintile when compared to sedentary individuals. A key finding presented in this study is that although a linear relationship between PA and adverse health outcomes was present, higher PA groups did not experience significantly greater benefits. Oja et al. [39] observed similar results in a cohort of British adults (30–98 years). After adjusting for a range of confounding factors, participation in vigorous activities was associated with significant reductions in the risk of all-cause mortality for some exercises in the study (cycling, swimming, racquet sports and aerobics) but not others (i.e.: running and football were not significant). Similar findings were present for CVD-specific mortality analysis with three activities associated with significant reductions in CVD-mortality (racquet sports, swimming and aerobics). Overall, these findings suggest that vigorous activities, particularly swimming, aerobics, and racquet sports, are beneficial for reducing mortality risks. Although running participation was not associated with a significant reduction in risk, these results do not contradict the beneficial effects of jogging/running on all-cause and CVD mortality.

Two studies investigated the relationship between prolonged PA and mortality in Danish populations [23, 43]. Schnohr [23] investigated this relationship using jogging as their exposure, finding that while joggers experienced lower rates of mortality, light and moderate joggers experienced the greatest benefits. Interestingly, strenuous joggers experienced similar mortality rates to non-joggers, with a U-shaped association suggesting that jogging 1 to 2.4 h per week at a light or moderate pace is most beneficial for longevity. The authors concluded that excessive running may not provide additional benefits and could diminish the advantages of moderate levels of jogging. Joseph et al. [43] observed an interaction between PA and blood pressure (BP) levels in relation to all-cause mortality. It was evident that an inverse dose-response relationship was present between PA and all-cause mortality for all BP levels, and that PA was associated with reductions in cardiovascular events, regardless of PA levels. A study on a Finnish population from the Helsinki Health Study by Lahti et al. [32] concluded that vigorous PA was associated with reduced mortality risk independently of the volume of leisure PA and other covariates. The association between intensity of PA and mortality were comparable for men and women.

Across the fourteen studies included in this review examining the relationship between high doses and intensity of PA and adverse cardiovascular outcomes, the protective nature of PA was universally visible. Four of these studies [23, 36, 38, 39] had results that were somewhat suggestive of identifying upper limits for the benefits associated with PA and a cut-off for a reduction in protective benefits (see Table 3).

**Table 3:**
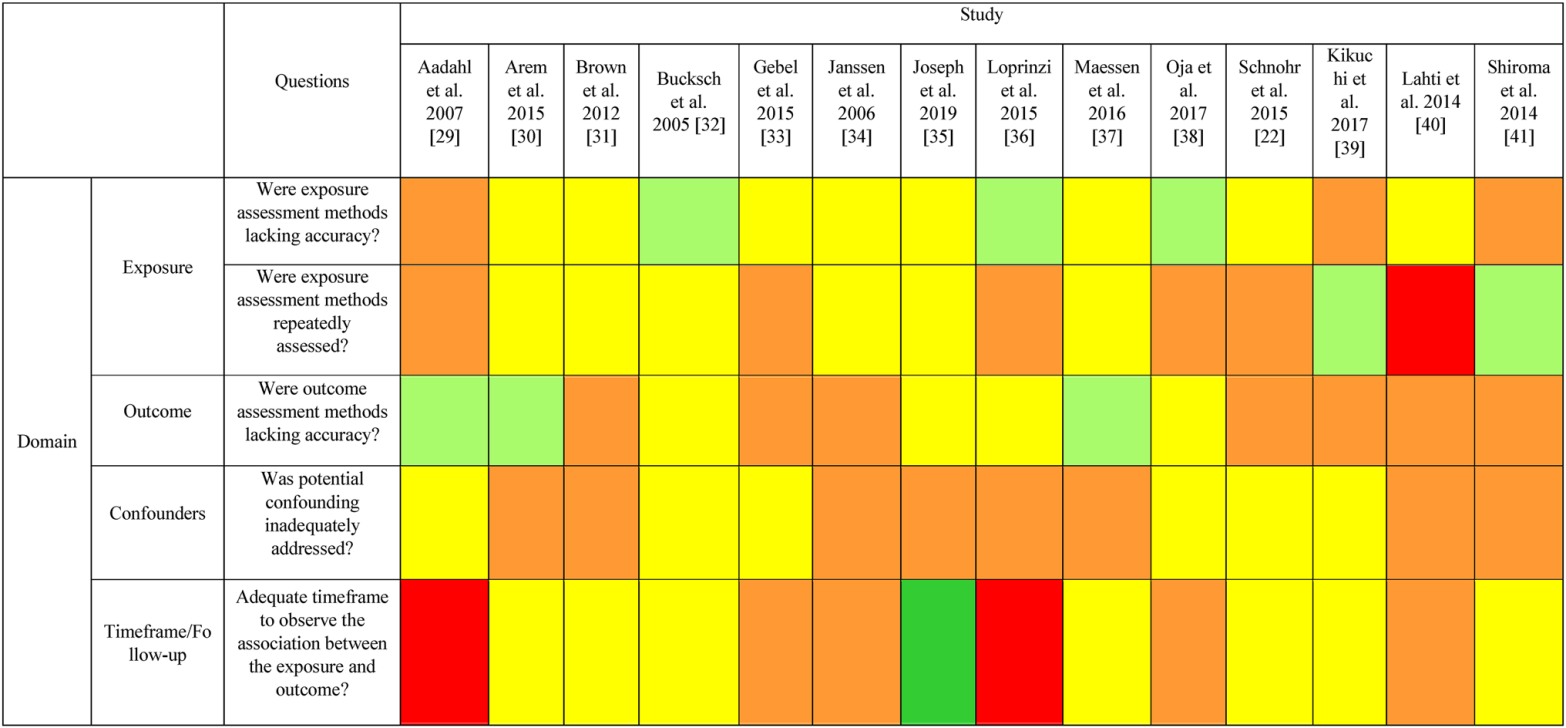
Risk of Bias in included studies assessed by adapted NIH quality assessment tool

Risk of Bias: Low Risk (Green), Probably Low Risk (Lighter Yellowish Green), Moderate (Yellow), Probably High (Orange), High Risk (Red)

## Discussion

The impact of light and moderate exercise on cardiovascular health has been extensively examined in health literature [46–48], whereas strenuous PA (both high intensity and high volume of exercise) has not been explored to the same extent. Prior to this study, examinations of relevant literature have resulted in a scientific statement review, meta-analysis and guideline recommendations pertaining to the association between heightened PA and cardiovascular health/mortality [8, 9, 27]. These reviews concluded that the benefits associated with PA would far outweigh the risks for the majority of those in the general population, whilst also not confirming a threshold for when the lifespan could be compromised [9, 49]. Our comprehensive review of relevant literature synthesises and critically evaluates evidence on the impact of strenuous exercise on the risk of adverse cardiovascular-related events and mortality which enables a more balanced assessment of evidence as previous reviews did not adequately explore the implications of PA classifications and measurements, timeframes, outcome and relevant confounding variables.

Across the included studies, most findings indicated that engaging in strenuous PA was either beneficial or neutral, with cardiovascular benefits levelling off at very high doses rather than continuing to increase. Two studies [23, 39] reported a U-shaped association, suggesting that strenuous PA was associated with a loss of cardioprotective benefit compared with more moderate doses. In both studies, the confidence intervals around these estimates were wide and not statistically significant, and the apparent patterns were difficult to interpret given the small number of participants engaging in strenuous PA, imprecise exposure classifications, and other methodological limitations identified in the risk of bias assessment. Overall, the broader evidence is more consistent with a plateauing of benefits, rather than a meaningful reversal of the protective effects of strenuous PA.

### Classification of strenuous exercise intensity/measurement of PA

There are a variety of ways that strenuous PA is defined and reported. None of the studies in this review incorporated objective measures when assessing PA, instead opting for self-reported measures. Self-reported PA is commonly utilised in population research due to its low cost and simple assessment methods [50]. Despite its cost-effectiveness and ease of use, self-reported PA has been identified as having low correlations with objective measures, large variability between different assessment tools, as well as both over- and under-estimations of true behaviours [51, 52]. Therefore, none of the studies in this review received a low risk of bias grading for this domain. Most studies incorporated METs (except two studies [34, 36]) when calculating total PA levels but varied when estimating PA intensity.

Grouping vigorous activities with the same MET was common in the studies in this review. The majority of the included studies [23, 33, 35, 36, 38, 43] assigned a single MET value for all of the vigorous activities that were included in assessment tools. This approach increases the potential risk of bias as activities above or below the MET thresholds used are under- or over-estimating the true output for PA and potentially misclassifying individuals to a low, moderate or vigorous group. Four studies [37, 39, 41, 42] used precise estimations of METs when classifying an individual’s PA intensity. These studies utilised Compendiums of Physical Activities to obtain accurate estimates for PA intensity, which were developed to quantify energy costs (METs) associated with a large variety of activities, which ultimately increase the comparability of results across studies using self-reported PA [53].

Although the PA Compendiums are useful in estimating intensities, the variation between self-perceived PA intensity and the actual rate at which the heart is being exerted is a potential source of bias. This has been explored in a previous study comparing objective and subjective measures of PA intensity by Skatrud-Mickelson et al. [54]. This study found that regular exercisers (similar to those included in this review) underestimated exertion during PA and that the true intensity of exercise was in fact higher [54]. The use of self-perceived intensity data from self-reported measures ultimately increase the potential for bias to be present for the studies in this review, with variability in PA intensity potentially resulting in incorrect classification of individuals to moderate or vigorous PA categories and/or miscalculations for total PA energy expenditure [55]. Further issues begin to arise pertaining to the appropriate cut-off point for vigorous activity due to the varying MET estimates and calculations for total PA energy expenditure (weekly METs, MVPA mins etc.). As the studies in this review varied with the cut-off points used for intensities of PA (METs) and the different categories for total weekly energy expenditure (MVPA/TEE), the likelihood of one or more occurring prematurely increases the risk of individuals being misplaced into moderate sub-groups instead of their vigorous counterparts [56, 57]. These differences make comparisons of these studies more difficult and increase the chance for over- or underestimations of the true effect of vigorous PA on health.

### Repeated PA measures/assessments

Repeated measurements help increase the reliability of exposure data, whilst also allowing researchers to investigate changes in behaviour over time [31, 58]. Janssen & Jollifee [36] was the only study in this review that measured PA behaviours more than once, with the authors assessing PA behaviours for the two weeks prior to examinations at both baseline and the initial three-year examination. This enabled the analysis of mortality risk relative to PA behaviours at both baseline and three-year follow-up, examining changes in PA behaviours. The inclusion of repeated PA assessments in these studies would have both strengthened the accuracy of PA data obtained from respondents, whilst also allowing the research groups to investigate whether changes in PA behaviour had an effect on outcomes and what levels of PA yielded the most protective benefits.

### Outcome assessments

The vast majority of the studies in this review [23, 32, 34, 37, 39, 41, 42, 46] included mortality as a primary outcome variable. These studies ascertained the death status of individuals through death certificates, national databases, medical records and probabilistically matching. Although each of these studies used standardised methods to obtain the mortality status of individuals, there was some variation in cause of mortality. Of the studies that incorporated all-cause mortality as an outcome variable, only two [39, 41] provided additional analyses delving into disease specific mortality and received a low risk of bias rating, with the remaining studies [23, 32–34, 36, 42] incorporating deaths from any causes. The studies that included mortality from all causes only as an outcome variable received a moderate risk of bias grading, as their underlying causes of death were not strictly cardiovascular in nature or related to other relevant risk factors and could have been completely unrelated to cardiovascular health and PA. Two studies in this review incorporated outcome measures other than mortality. Maessen et al. [38] analysed the relationship between PA behaviours and CVD and the presence of cardiovascular risk factors (the presence of hypertension, hypercholesterolemia and/or type two diabetes), confirming an individual’s classification to either group against the medications they took. Aadahl et al. [35], took a similar approach but instead measured a range of biological variables during clinical examinations. Each of these two studies received a low risk of bias grading due to the standardised methods used to assess relevant risk factors for adverse cardiovascular events and mortality. The remaining studies [37, 43] included both mortality and a cardiovascular variable as outcome variables. Although the inclusion of cardiovascular biomarkers [37] and cardiovascular events [43] helped to provide a better understanding of the association between PA and cardiovascular outcomes using standard protocols, both studies included all-cause mortality. Consequently, these studies both received a moderate risk of bias grading as relevant cardiovascular data was included, but mortality was from all causes and not disease- or cardiovascular-specific.

### Timeframe (follow-up)

As highlighted in the introduction, there are various clinical pathways through which PA can lead to a cardiovascular event, but a common pathway is that long-term excessive endurance exercise may induce pathologic structural remodelling of the heart and large arteries [22]. Therefore, a substantial timeframe is required to observe PA levels and cardiovascular/mortality outcomes. Two studies [35, 37] in this review were limited in their examination of the true effect of their exposure on their outcome(s) due to their cross-sectional components of their data collection, ultimately resulting in a high likelihood of bias [59]. For three of the cohort studies [34, 36, 39], the time for follow-up increased the potential for bias, as participants were followed over an average timeframe less than ten years. Six studies [23, 33, 40–42, 44] in this review had a timeframe for follow-up between ten and twenty years with a lower potential for bias. Two studies in this review [38, 43] had follow-ups that were longer than twenty years. Despite this, the study by Maessen et al. [38] has a high probability of bias as the timeframe for follow-up was based on recall data. Lifelong exercise patterns in this study were assessed by asking participants to recall their exercise habits across four life periods. Although participants were able to provide sufficient data, recall bias could be present and influence the accuracy of PA data provided. The timeframe (mean 23.4 ± 11.7 years) used by Joseph et al. [43] allows for the effects of high doses and intensities of PA to occur, and any associated adverse cardiovascular events to take place. This is the only study in this review that has received a low risk of bias grading. Greater timeframes to examine the effects of PA on cardiovascular/mortality outcomes are needed to reduce the potential for bias in the majority of the studies in this review.

### Confounders/covariates

Incorporating relevant confounding factors increases confidence in establishing real relationships and contributes to the internal validity of the research. When examining the relationship between vigorous PA and cardiovascular risk factors, as well as mortality, there is a necessity to control for body weight, smoking status, dietary intake as well as blood pressure and blood cholesterol, as they are associated with adverse cardiovascular outcomes and mortality [4, 60]. In addition to these prominent risk factors, further adjustments need to be made with regard to sociodemographic variables (age, sex, race, education, marital status, residence, SES) and chronic disease status due to independent associations with overall health and wellbeing, cardiovascular events and mortality risk [61–63].

Overall, controlling for confounding in the statistical analyses varied for the fourteen studies in this review. All of the studies in this review allowed for potential confounders such as age and sex of respondents, which is standard practice in health research due to the independent effects of each variable on both general and cardiovascular-specific health [64, 65]. Age is a well-established cardiovascular risk factor, with older adults experiencing substantially higher rates of cardiovascular events and mortality than younger individuals due to age-related physiological changes and accumulation of risk factors over time [66]. Age may therefore act as an important effect modifier in the relationship between strenuous PA and cardiovascular outcomes; however, none of the included studies formally examined age-stratified effects or tested for interaction. Smoking status was incorporated into all studies excluding one [37], as it would have moderately reduced the total sample size for this study. Both level of education and marital status are commonly used as confounding variables, however, level of education was used in seven studies [23, 33, 34, 38, 39, 41, 43], whereas marital status was only present in three studies [33, 34, 41]. A variety of other sociodemographic variables were scarcely used across the fourteen studies in this review including race [36, 37], social class [42], urban or rural residency [34] as well as socioeconomic status and self-perceived health status [36].

Further adjustments for health-related variables were common in the studies for this review. Body mass/composition is a variable often used in health research as a confounding factor due to its relationship with adverse health [67]. Additionally, obesity has been established as a key risk factor for the development of coronary heart disease (CHD), as well as a risk factor for many prominent detrimental cardiovascular health outcomes such as hypertension and dyslipidaemia [68]. Measurements of body mass, weight and/or adiposity were incorporated into all but two studies in this review [23, 38] and although there are limitations of measuring body composition with BMI, it has been proven to correlate with body fat, future health risks, morbidity and death [69]. Consequently, both of these studies would have a higher likelihood of bias.

The presence/absence of chronic health conditions is another key variable when examining the relationship between PA and cardiovascular health due to its association with poorer health outcomes and likelihood of comorbidities [70]. Confounding for the presence or history of chronic conditions was completed by most of the studies in this review, with only three studies not accounting for this [35, 37, 39]. For those studies examining chronic conditions, the number of conditions varied between studies ranging from one [23], two [41] or four or more [33, 34, 36, 38, 42, 43]. Therefore, an increased potential for bias was noticed not just in the studies that did not account for chronic health conditions [35, 37, 39], but also Schnhor et al. [23] as the authors only adjusted for diabetes. The variability in accounting for cardiovascular-related diseases suggests that findings from these studies could differ significantly according to the specific conditions included, emphasizing the need for more comprehensive and standardised confounding adjustments to accurately assess the impact of PA on long-term cardiovascular health and mortality.

Adjusting for measured biomarkers was notably limited among the studies in this review. While three studies utilised biomarkers as outcome variables [35, 37, 38], four studies accounted solely for blood pressure as a confounding variable [23, 36, 40, 43]. This limited inclusion, as well as the absence of confounding in the remaining seven studies, significantly increases the potential for bias, as it restricts these studies’ ability to account for the physiological pathways linking PA with cardiovascular health outcomes. While the collection of biomarker data in large-scale public health studies poses considerable challenges due to the substantial costs involved [71], it is useful to provide a clearer understanding of the relationship between PA and cardiovascular health. To enhance the accuracy and depth of future research, efforts should prioritize the inclusion of clinically measured biomarkers as confounders when possible.

As previously mentioned, one of the most crucial variables for cardiovascular health and mortality is an individual’s dietary behaviours, which are regarded as a key risk factor for CVD and mortality [72–74]. Epidemiological research has identified that regardless of PA status, engaging in ‘healthy’ dietary behaviours is beneficial to health and longevity [75, 76]. Additionally, a recent study by Ding et al. [77] has concluded that although independent engagement in PA and positive dietary behaviours is beneficial to health, adhering to both is optimal for reducing the risk of adverse health outcomes. In a study of 346,627 individuals from the UK Biobank, the lowest risk of CVD- and activity, diet and adiposity-related cancer mortality was witnessed among individuals with the highest diet quality and PA level [77].

Of the studies in this review, controlling for nutritional behaviour and dietary intake was generally inadequate. Five of the studies in this review did not include a dietary variable as a confounder when completing statistical analysis [36–38, 43, 44]. Of the remaining studies, four controlled only for alcohol intake [23, 33, 39, 41] and three controlled for a combination of alcohol consumption and other dietary behaviours [34, 35, 42]. Similar to the studies above, Aadahl et al. [35] and Gebel et al. [34] recorded alcohol intake in drinks per week, whereas Bucksch [42] opted for daily consumption, then categorising individuals into three different groups (low, risky and dangerous). Fruit and vegetable intake was included in each of these studies to assess diet quality and has been shown to reduce the risk of CVD and mortality [78, 79]. Although fruit and vegetable intake alone are not an adequate measure of total diet quality, this measure is often used as a proxy for good health and is positively associated with diet score [80]. Aadahl et al. [35] used a 48-hour food frequency questionnaire to assess dietary behaviours and created “unprudent”, “moderately prudent” and “prudent” categories to distinguish their sample. Bucksch [42] assessed dietary intake based on the frequency that respondents ate breakfast (rarely vs. frequently) and positive dietary habits index (1–5, 6 and 7, 8 or more). Gebel and colleagues’ [34] 45 & Up questionnaire included a range of items to assess dietary behaviours; however, only combined fruit and vegetable consumption was incorporated into the analysis. The inclusion of these confounding variables in each study helped to reduce the likelihood of bias, however, the lack of holistic dietary assessments reduces the accuracy of diet quality classifications. Further, none of these studies incorporated assessments of sugar or sodium intake into analyses, which are associated with CVD, mortality and relevant risk factors [81, 82]. The lack of adequate confounding for nutritional behaviours and dietary intake increases the potential that the true effect of PA on cardiovascular health and mortality is uncertain in the fourteen studies included in this review, but more so for the eight studies with no nutrition/dietary assessment whatsoever.

### Limitations and recommendations of the review

Some limitations were present in this review, one of which was the lack of studies utilising Asian and African participants (with the exception of one study on a Japanese population [44]). Although each of the fourteen studies ranged with regard to geographical context and age of participants, limited data was available from non-western countries, meaning the results are not easily generalisable to individuals from these countries. Additionally, it remains uncertain whether the apparent plateau in benefits at very high volumes of PA reflects a true biological ceiling or is instead a result of healthy participant clustering, which cannot be examined in the existing evidence. It was also not possible to conduct a meta-analysis (and compare or pool results) as many of the studies were not comparable due to many of the issues identified previously (e.g. measurement of PA, timeline involved and measurement of outcome).

For each of the issues identified in the studies for this review we have mentioned how they could be better addressed. Overall, a key recommendation is better designed longitudinal cohort studies or case-control studies within or separate to these studies with a particular focus on strenuous PA over a longer period of time and an ability to control or allow for many of the issues raised in this review (e.g. need for longer time frames, control for confounding variables, etc.).

## Conclusion

This review has assessed relevant literature regarding the relationship between strenuous (high volume and intensity) PA and cardiovascular outcomes. The majority of findings suggest that prolonged strenuous PA is not harmful to cardiovascular health, but there is some evidence that the benefits may plateau at some point. Only two studies suggested a U-shape relationship whereby additional exercise may be harmful, but both studies suffered from a number of methodological issues with the potential for a high risk of bias. Furthermore, all of the studies in this review had sources of potential bias across multiple domains that reduce their strength/validity. Overall, it was clear that any level of strenuous PA was beneficial to cardiovascular health and that little-to-no further benefits were present for individuals greatly exceeding PA recommendations compared to those who did not. Public health messaging and recommendations need to reflect this to further address physical inactivity but stress the importance for at-risk individuals to maintain caution and progressively increase PA intensity and loads.

## Supplementary Information

Below is the link to the electronic supplementary material.

Supplementary material 1 (DOCX 113.8 kb)

## Data Availability

The data supporting the findings of this study are available within the article and its supplementary materials. Further data information can be provided from the author upon reasonable request. We registered our systematic review in the Open Science Framework Registries with the following identifiers: (1) Associated project: https://osf.io/v2z8p (2) Registration DOI (available after embargo is lifted) – anonymised view-only link is available for review: https://osf.io/4g95s/overview? view_only=18bd2b6998dc4a5d996a0a9ba288ff45.
